# Retrospective Analysis of the Clinical Outcome in a Matched Case-Control Cohort of Polytrauma Patients Following an Osteosynthetic Flail Chest Stabilization

**DOI:** 10.3390/jcm9082379

**Published:** 2020-07-26

**Authors:** Marcel Niemann, Frank Graef, Serafeim Tsitsilonis, Ulrich Stöckle, Sven Märdian

**Affiliations:** Center for Musculoskeletal Surgery, Charité–University Medicine Berlin, Augustenburger Platz 1, 13353 Berlin, Germany; frank.graef@charite.de (F.G.); serafeim.tsitsilonis@charite.de (S.T.); ulrich.stoeckle@charite.de (U.S.); sven.maerdian@charite.de (S.M.)

**Keywords:** Polytrauma, flail chest, thoracic injury

## Abstract

Background: In polytrauma (PT) patients, osseous thoracic injuries are commonly observed. One of the most severe injuries is the flail chest where the rib cage is broken in such a way that leads to a partial functional detachment of the thoracic wall. Especially in PT patients, the integrity of the respiratory system and especially, of the respiratory muscles is essential to prevent respiratory failure. Besides conservative treatment options, flail chest injuries may be surgically stabilized. However, this treatment option is rarely carried out and evidence on the outcome of surgically treated flail chest patients is rare. Objective: This study intends to investigate the clinical outcome of PT patients with the diagnosis of a flail chest who received an osteosynthetic stabilization for that compared to the same group of patients without an operative treatment. The between-groups outcome was compared regarding the duration of the total hospital and the intensive care unit (ICU) stay, the total of the invasive ventilation days, the incidence of pneumonia, and the dosage of the pain medication at the hospital discharge. Methods: A retrospective analysis was conducted including all PT patients who received an osteosynthetic stabilization of a flail chest. Furthermore, another cohort of PT patients and the diagnosis of a flail chest but without operative treatment was determined. Both groups were case-control matched for the Injury Severity Score (ISS) and age. Further statistical analysis was performed using the Wilcoxon signed-rank test and the McNemar’s test. Results: Out of eleven operatively and 59 conservatively treated patients, eleven patients per group were matched. Further analysis revealed no significant differences in the normal ward treatment duration (5.64 ± 6.62 and 6.20 ± 5.85 days), the invasive ventilation duration (was 6.25 ± 7.17 and 7.10 ± 6.14 days), the morphine equivalent dosage of the oral analgesia (61.36 ± 67.23 mg and 39.67 ± 65.65 mg), and the pneumonia incidence (36.4 and 54.5%) when conservatively and operatively treated patients were compared, respectively. However, surgically treated patients had a longer ICU (25.18 ± 14.48 and 15.27 ± 12.10 days, *Z* = −2.308, *p* = 0.021) and a longer total hospital treatment duration (30.10 ± 13.01 and 20.91 ± 10.34 days, *Z* = −2.807, *p* = 0.005) when compared to conservatively treated patients. Conclusion: In the present study cohort, there was no outcome difference between conservatively and operatively treated patients with the diagnosis of a flail chest regarding the normal ward treatment duration, the invasive ventilation duration, the morphine equivalent dosage of the oral analgesia, and the pneumonia incidence while ICU treatment duration and hospital treatment duration was longer in operatively treated patients.

## 1. Introduction

In 2018, the TraumaRegister^®^ DGU counted 17,664 severely injured trauma patients, who were admitted to a hospital in Germany. Out of these, 4735 patients met the criteria of a polytrauma (PT) [1].

In PT patients, severe injuries of the thorax are regularly observed. At least 53.2% of the PT patients after a fall from a height [2], 42.8% of the pedestrians, and 60.1% of the motor vehicle occupants with a blunt trauma [3] presented with a severe injury of the thorax with an Abbreviated Injury Scale (AIS) [4] ≥ 3. Osseous injuries of the thorax are associated with a high level of pain [5] and, potentially, with a longer hospital stay and a prolonged invasive ventilation, especially when associated with sternal fractures [6]. Accordingly, a surgical stabilization of flail chest injuries might entail avoiding such issues. Yet, evidence of the benefits on patient outcome following surgical flail chest treatment is rare.

There is one meta-analysis comparing three randomized controlled trials (RCTs) of traumatic flail chests which concludes that surgical stabilization of a flail chest leads to a lower incidence of pneumonia, a shorter duration of mechanical ventilation, and a shorter length of intensive care unit (ICU) stay [7]. The authors of the primary studies used heterogenous inclusion criteria, surgical indications, and surgical methods [8,9,10] which might be the reason for the data heterogeneity observed by the authors of the meta-analysis [7].

Therefore, we conducted a retrospective study with a case-control matched cohort of PT patients and the diagnosis of a flail chest. Uniquely, there was no exclusion of patients because of any associated injury. Patients who received a surgical stabilization of the rib cage were compared to a conservative treatment group. The between-groups outcome was compared regarding the length of the hospital and the ICU stay, the invasive ventilation days, the incidence of pneumonia, and the dosage of the pain medication at the hospital discharge.

## 2. Methods

A retrospective analysis was conducted between January 2012 and December 2019 concerning all patients transmitted to the Campus Virchow clinic of the Center for Musculoskeletal Surgery of the Charité–University Medicine Berlin, a German level 1 trauma center. The electronic medical data system used at this clinic, SAP (SAP ERP 6.0 EHP4, SAP AG, Walldorf, Germany), was searched for PT patients in the aforementioned time span. PT patients were defined as patients whose clinically and radiographically objectified injuries counted up to an ISS ≥ 16. Therefore, the recorded diagnoses codes of the 10th version of the International Classification of Diseases (ICD-10) and the injuries described in the radiology reports were manually translated to their specific AIS codes in the version of 2005. The Injury Severity Score (ISS) was calculated by adding up the squares of the AIS codes of the three most injured body regions [11]. Furthermore, regarding the already described limitations of such scoring systems in patients with injuries of one region, solely [12], it was decided to include patients as previously described only when matching to the ‘Berlin Definition’ [13], as well. Patients who preclinically died or passed away after hospital but before ICU admittance were excluded from any further analysis.

Out of these patients, those with a flail chest according to the documented clinical and radiographic findings were included in the subsequent analysis. Flail chest was defined as either at least three contiguous ribs with segmental fractures or more than five adjacent rib fractures. All patients meeting the described criteria were included in the definite analyses. Demographic data were assessed, such as sex, age, the dates of hospital admittance, ICU discharge, and hospital discharge and morphine equivalent dosage at hospital discharge.

During the hospital stay, some of the included patients required secondary osteosynthetic stabilization of their rib cage. The indication for that intervention was made in a multidisciplinary approach both with trauma surgeons and intensive care physicians. Surgery was planned only if both disciplines agreed on the necessity of a surgical chest wall stabilization. At our clinic, PT patients with the radiographic diagnosis of a flail chest were selected for surgery when they presented a clinically observed paradoxical breathing pattern that lead to a prolonged weaning process. A paradoxical breathing pattern was defined as a flail segment moving inward due to the negative pleural pressure during regular inspiration while the rest of the thoracic wall physiologically moved outward [14]. A prolonged weaning process was defined as a futile extubation attempt ≥48 h or the necessity of a re-intubation within 48 h and, in the meantime, an observed paradoxical breathing pattern.

At our clinic, the MatrixRib™ system (DePuy Synthes, Raynham, MA, USA) was used for such operations. Commonly, this intervention was performed by an anterolateral thoracotomy. Following an exploration of the thoracic cavity and, depending on the injuries found, appropriate interventions such as evacuation of a hemothorax, coagulation of an active intrathoracic bleeding or overhaul of injured lung parenchyma, the rib cage was reconstructed. Therefore, the ribs beyond and above the thoracotomy as well as another more cranial rib were reduced and stabilized using plates out of the aforementioned osteosynthetic system. Furthermore, a thoracic drainage was inserted and the operation site was closed in a usual manner using non-resorbable sutures.

In order to avoid confounding when comparing conservatively and operatively treated patients with a flail chest, a case-control matching in the mentioned subgroups was performed. Tolerance limits were set as ± four and ± five and, correspondingly, patients were matched for ISS [15] and for age [16], respectively, in a ratio of 1:1.

The statistical analysis was performed using SPSS (SPSS Statistics for Mac OS, Version 25, IBM Corp., Armonk, NY, USA). Initially, the implemented case-control matching was used to create a matched case-control cohort of conservatively and operatively treated flail chest cases in the aforementioned manner. The resulting data were analyzed for normal distribution using histograms, Q-Q plots, and the Shapiro–Wilk test. Accordingly, the Wilcoxon signed-rank test and the McNemar’s test were used for dependent samples. Unless stated otherwise, values are represented as mean ± SD. All *p*-values are two-tailed and *p*-values ≤ 0.05 were considered statistically significant.

## 3. Results

### 3.1. Demographic Data of the Study Cohort

Regarding the described inclusion criteria, a total of 70 PT patients (17 females, 53 males) with a flail chest were included in the analysis. The entire demographic data of the study cohort are displayed in Table 1.

In the conservative treatment group, 13 (22.0%) patients were regularly sent home, twelve (20.3%) patients were discharged to an intensive care rehabilitation program, and 28 (47.5%) patients were discharged to a regular care rehabilitation program. In the operative treatment group, three (27.3%) patients were regularly sent home, three (27.3%) patients were discharged to an intensive care rehabilitation program, and four (36.4%) patients were discharged to a regular care rehabilitation program.

In the conservative treatment group, six patients (10.2%) died during the primary hospital stay. One patient (1.7%) died of a pulmonary embolism, three patients (5.1%) of a post-hypoxic cerebral edema >24 h after hospital admittance, and in two patients (3.4%), a palliative therapy was multidisciplinarily chosen after the primary computed tomography scan. In the operative treatment group, one patient (9.1%) died of a pulmonary embolism.

### 3.2. Demographic Data of the Case-Control Matched Cohort

Following the case-control matching, eleven patients (3 females, 19 males) remained in the conservative treatment group. Those were matched with the operative treatment group. None of the conservatively treated patients died during the primary hospital stay, but one surgically treated patient (male, 52 years old, ISS 20) died of a pulmonary embolism at day 18 after the primary hospital admission. The demographic data of the case-control matched cohort are represented in Table 2. There were no surgical site complications noted during the hospital stay.

In the conservative and in the operative treatment group, four (36.36%) and three (27.27%) patients were regularly sent home, two (18.18%) and three (27.27%) patients were discharged to an intensive care rehabilitation program, and five (45.45%) and four (36.36%) patients were discharged to a regular care rehabilitation program, respectively.

### 3.3. Outcome Analysis of the Case-Control Matched Cohort

While patients of the conservative treatment group stayed at the hospital for 20.91 ± 10.34 days (range 7.0–36.0 days), patients of the operative treatment group stayed for 30.10 ± 13.01 days (range 12.0–54.0 days). This difference was statistically significant (*Z* = −2.807, *p* = 0.005).

During that time, conservatively treated patients stayed at the ICU for 15.27 ± 12.10 days (range 1.0–36.0 days) and at the normal ward for 5.64 ± 6.62 days (range 0–17.0 days). Operatively treated patients stayed at the ICU for 25.18 ± 14.48 days (range 5.0–48.0 days) and at the normal ward for 6.20 ± 5.85 days (range 0–15.0 days). The difference in the ICU treatment duration significantly differed between groups (*Z* = −2.308, *p* = 0.021), while the difference of the normal ward treatment duration did not reach significance between groups (*Z* = −1.054, *p* = 0.292).

During the ICU stay, patients conservatively treated stayed for 6.25 ± 7.17 days (range 0–20.0 days) and patients operatively treated stayed for 7.10 ± 6.14 days (range 0–19.0 days). The differences between groups did not significantly differ (*Z* = −1.192, *p* = 0.233).

At the time of hospital discharge, conservatively treated patients needed an equivalence of 61.36 ± 67.23 mg (range 0–195.0 mg), while operatively treated patients needed an equivalence of 39.67 ± 65.65 mg (range 0–180.0 mg) oral morphine as a pain medication. The differences between groups did not reach significance (*Z* = −0.845, *p* = 0.398).

A total of four patients (36.4%) out of the conservatively treated and six patients (54.5%) out of the operatively treated group received antibiotic treated due to the clinical and radiographic diagnosis of a pneumonia. This difference did not reach significance (*p* = 0.687).

**Figure 1:**
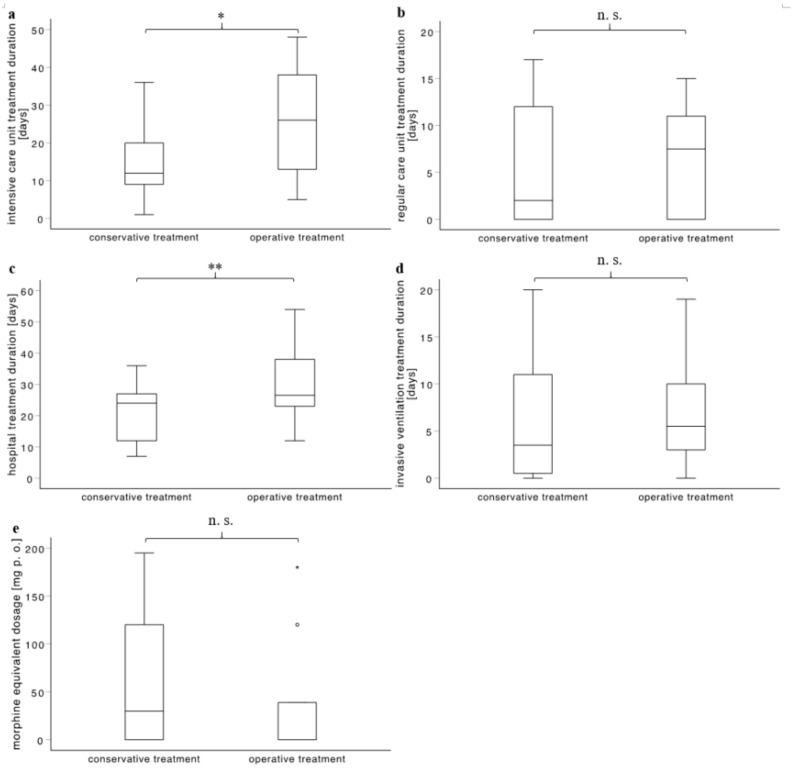
Overview of the outcome of the case-control matched cohort. (**a**) depicts the duration of the intensive care unit treatment duration which was 15.27 ± 12.10 and 25.18 ± 14.48 days in conservatively and operatively treated patients, respectively (*Z* = −2.308, *p* = 0.021). (**b**) depicts the duration of the normal ward treatment duration which was 5.64 ± 6.62 and 6.20 ± 5.85 days in conservatively and operatively treated patients, respectively (*Z* = −1.054, *p* = 0.292). (**c**) depicts the total hospital treatment duration which was 20.91 ± 10.34 and 30.10 ± 13.01 days in conservatively and operatively treated patients, respectively (*Z* = −2.807, *p* = 0.005). (**d**) depicts the duration of the invasive ventilation which was 6.25 ± 7.17 and 7.10 ± 6.14 days in conservatively and operatively treated patients, respectively (*Z* = −1.192, *p* = 0.233). (**e**) depicts the pain medication at the time of hospital discharge as morphine equivalent dosage. This was 61.36 ± 67.23 mg and 39.67 ± 65.65 mg in conservatively and operatively treated patients, respectively (*Z* = -0.845, *p* = 0.398). * *p* ≤ 0.05, ** *p* ≤ 0.01, n. s. *not significant.*

Figure 1 displays the outcome of the case-control matched cohort regarding the intensive care unit treatment duration, the regular ward treatment duration, the total hospital treatment duration, the invasive ventilation treatment duration, and the morphine equivalent dosage at hospital discharge.

## 4. Discussion

The presented study investigated clinical outcome differences in a matched case-control cohort of PT patients with the diagnosis of a flail chest. While the normal ward treatment duration, the invasive ventilation duration, the morphine equivalent dosage of the oral analgesia at the time of hospital discharge, and the pneumonia incidence did not significantly differ between groups, the ICU and the total hospital treatment duration were significantly longer when the flail chests were surgically stabilized.

Granhed and Pazooki provided a prospective cohort study including 60 patients who received a chest wall stabilization due to a flail chest. The definition of the flail chest as an indication for surgery and the surgical procedure were very similar to the ones mentioned above. The authors compared that cohort with historical controls who did not receive surgery and found a significantly shorter invasive ventilation duration for operated patients [15]. This is supported by some authors who had similar results [8], while others did not find any significant difference between groups [10]. However, Granhed and Pazooki did not match for the ISS which was 30.9 ± 13.3 in the historical cohort compared to 21.7 + 10.7 in the primary analyzed cohort. The authors claimed that the ISS did not significantly differ between groups. Regardless of that, they found a linear correlation between the ISS and the invasive ventilation duration [15]. Therefore, the different ISS, although not reaching significance, should be discussed as a potential confounder of the published results.

Nevertheless, the different results of previous authors [8,15] when compared to our results might be a result of the point of time when surgery took place. While other authors reported an early intervention, at our department the indication for a surgical intervention in flail chest patients is made when other conservative treatment options repeatedly failed. This decision is made in the manner of a last treatment option. This needs to be taken into account when comparing our results to previously published data.

Farquhar et al. provided a retrospective cohort of 19 patients with a mean ISS of 31 who received surgery for a flail chest injury. The included patients were matched for the AIS of the thoracic injury, their age, and the AIS of the other injured regions with a historical cohort of conservatively treated patients with a mean ISS of 29. The authors found significantly less invasive ventilation days, a shorter ICU and hospital stay, and lower rates of pneumonia in the control group when compared to the surgically treated group [17]. Other authors, on the other hand, found a lower pneumonia incidence and a shorter ICU treatment duration [8,10] as well as a shorter total hospital treatment duration [10] in surgically treated when compared to conservatively treated patients.

Another recent study by Caragounis et al. analyzed a prospective consecutive series of 49 patients surgically treated due to a flail chest. The authors found a decreasing pain level as well as an improving forced vital capacity (FVC) during the observation period of one year. However, the authors excluded patients with severe head injury or any spinal cord injury which, as already described above, might confound the cohort when PT patients are considered [18]. In the presented study, the morphine equivalent dosage of the oral analgesia at the time of hospital discharge appeared to be lower following surgery but the difference between groups did not reach significance. This might be a result of the small study cohort. Olsén et al. provided a cohort of 31 prospectively acquired patients with a mean ISS of 22 and the diagnosis of a flail chest who received surgical stabilization. These were compared to 30 unmatched historical patients with a mean ISS of 18.5 who were treated conservatively for the same diagnosis. The authors did not find any significant differences regarding pain and FVC in the follow-up [19].

The currently available literature about the surgical stabilization of a flail chest is heterogenous regarding the indication, the material used, the patient groups themselves, and the type of statistical analysis. Concurrently more data in a RCT-design needs to be published in order to derive general recommendations regarding the aforementioned intervention. Especially, the timing of a surgical intervention for a flail chest in PT patients needs to be investigated. When used as a last treatment option after conservative treatment failure, surgical flail chest stabilization cannot facilitate any benefits regarding the outcomes observed in this study.

Perchance, algorithms such as the one provided by Bemelmann et al. [20] need to be taken into account when such future studies are planned in order to generate some kind of standard operating procedure.

Up until now, previous studies proved that several biomarkers are associated with infectious complications as well as general outcome after polytrauma [21,22]. Moreover, authors were able to show that fracture healing is connected with individual’s immune reaction [23,24,25]. Correspondingly, the interactions between the immunoinflammatory mechanisms in polytrauma patient with associated osseous thoracic injuries should be addressed in future study designs as well.

This study has both strengths and limitations. Firstly, the study was designed in a matched-case control design including a matching for commonly known confounders. This effectively reduces selection bias. However, both groups are not totally identical. Patients of the operatively treated cohort had a bilateral flail chest more often than patients of the conservatively treated group. Although not adequately represented in the ISS, a bilateral flail chest might potentially compromise the physiological breathing mechanisms even more. Secondly, there was no patient exclusion because of any related injuries. In such fashion, the general cohort of PT patients is adequately represented in this study. On the other hand, the overall study population was low considering a total of 22 patients that could matched. Future studies need to be designed to investigate a larger population in order to generate more evidence for recommendations. Also, the severity of the thoracic injuries differed between groups. In the conservative treatment group, all patients solely had a unilateral flail chest. In the operative treatment group, only two patients had a unilateral flail chest, while nine patients had a bilateral flail chest. This might compromise the patients’ outcomes and, therefore, needs to be taken into account when comparing this study results to previously published data. Even though case-control matched, the groups might not be completely identical. Lastly, a surgical chest wall stabilization was chosen as a last treatment option. Correspondingly, operatively treated patients already stayed longer at the ICU when the definite treatment procedure was chosen. This needs to be taken into account when interpreting the presented data.

## 5. Conclusions

In the present study, there were no significant differences between flail chest patients who were operatively treated when compared to conservatively treated patients regarding the length of the regular ward stay, the invasive ventilation days, the morphine equivalent dosage at the time of hospital discharge, and the pneumonia incidence. The ICU and the general hospital treatment duration were longer in the surgically treated group.

However, neither from the presented nor from the already published data can a general recommendation be derived. Especially in PT patients, data are lacking. There is a high need for RCTs in order to generate such data. Until then, the indication for surgical stabilization of flail chest injuries might remain as a last treatment option.

## Figures and Tables

**Table 1:** Demographic overview of the study cohort.

	Total Sample (N = 70)	Conservative Treatment (N = 59)	Operative Treatment (N = 11)
sex (female/male)	17/53	17/42	0/11
age [years]	50.69 ± 16.18 (range 16–82)	51.46 ± 16.25 (range 16–81)	46.55 ± 15.85 (range 29–82)
ISS	33.24 ± 12.17 (range 16.0–75.0)	33.97 ± 12.07 (range 16–75)	29.36 ± 12.55 (range 16–57)
Flail chest (unilateral/bilateral)	50/20	48/11	2/9
Pneumonia (no/yes)	39/31	34/25	5/6
Morphine equivalent dosage [mg p. o.]	100.65 ± 236.95 (range 0–1200.0)	111.0 ± 253.87 (range 0–1200.0)	39.67 ± 65.65 (range 0–180.0)
Hospital treatment [days]	23.90 ± 13.69 (range 1.0–68.0)	22.85 ± 13.63 (range 1.0–68.0)	30.10 ± 13.01 (range 12.0–54.0)
Intensive care unit treatment [days]	17.91 ± 14.59 (range 1.0–68.0)	16.56 ± 14.33 (range 1.0–68.0)	25.18 ± 14.48 (range 5.0–48.0)
Normal ward treatment [days]	6.28 ± 7.61 (range 0–35.0)	6.29 ± 7.91 (range 0–35.0)	6.20 ± 5.85 (range 0–15.0)
Invasive ventilation [days]	7.93 ± 9.10 (range 0–42.0)	8.17 ± 9.85 (range 0–42.0)	7.10 ± 6.14 (range 0–19.0)

ISS injury severity score, p. o. per os.

**Table 2:** Demographic overview of the case-control matched cohort.

	Conservative Treatment (N = 11)	Operative Treatment (N = 11)
sex (female/male)	3/8	0/11
age [years]	46.18 ± 13.78 (range 25–77)	46.55 ± 15.85 (range 29–82)
ISS	29.27 ± 11.58 (range 20–57)	29.36 ± 12.55 (range 16–57)
Flail chest (unilateral/bilateral)	11/0	2/9
Pneumonia (no/yes)	7/4	5/6

ISS injury severity score, p. o. per os.
